# Multidimensional perfectionism and orthorexia: a systematic review and meta-analysis

**DOI:** 10.1007/s40519-024-01695-z

**Published:** 2024-10-10

**Authors:** Verity B. Pratt, Andrew P. Hill, Daniel J. Madigan

**Affiliations:** 1 School of Science, Technology, and Health, York St John University, York, UK; 2 Graduate Department of Kinesiology, University of Toronto, Toronto, Canada

**Keywords:** Disordered eating, Weight, Diet, Psychopathology

## Abstract

**Purpose:**

We provide the first systematic review and meta-analysis of research examining multidimensional perfectionism—perfectionistic strivings and perfectionistic concerns—and orthorexia.

**Methods:**

The systematic review and meta-analysis was pre-registered and conducted using a search of PsycINFO, MEDLINE, Education Abstracts, and Oxford Academic, and ScienceDirect up to April 2023. PRISMA guidelines were also followed. Meta-analysis using random-effects models was used to derive independent and unique effects of perfectionism, as well as total unique effects (TUE), and relative weights. Moderation of effects were examined for age, gender, domain, perfectionism and orthorexia instruments, and methodological quality.

**Results:**

Eighteen studies, including 19 samples (*n* = 7064), met the eligibility criteria with 12 of these studies (with 13 samples; *n* = 4984) providing sufficient information for meta-analysis. Meta-analysis revealed that perfectionistic strivings (*r*^+^  = 0.27, 95% CI [0.21, 0.32]) and perfectionistic concerns (*r*^+^  = 0.25, 95% CI [0.18, 0.31]) had positive relationships with orthorexia. After controlling for the relationship between perfectionism dimensions, only perfectionistic strivings predicted orthorexia which also contributed marginally more to an overall positive total unique effect of perfectionism (TUE = 0.35; 95% CI [0.28, 0.42]). There was tentative evidence that orthorexia instrument moderated the perfectionistic concerns-orthorexia relationship.

**Discussion:**

Research has generally found that both dimensions of perfectionism are positively related to orthorexia. More high-quality research is needed to examine explanatory mechanisms while also gathering further evidence on differences in findings due to how orthorexia is measured, as well as other possible moderating factors.

**Level of evidence:**

Level 1, systematic review and meta-analysis.

**Supplementary Information:**

The online version contains supplementary material available at 10.1007/s40519-024-01695-z.

## Introduction

A healthy lifestyle is essential for a long and happy life. However, the promotion of a healthy lifestyle can become problematic if accompanied by rigid and unrealistic messages [1]. With this in mind, a desire for good health can, for some, be supplanted by an obsession with a desire for “perfect health”—perfect health choices, perfect bodies, and perfect eating habits. This danger is apparent in popular media and social media platforms, in particular, which often promote unrealistic health ideals, images, and lifestyles [2]. Orthorexia—characterised by some as the pursuit of the “perfect diet” [3]—is gaining interest among researchers and practitioners, and has recently been linked to an individual’s general perfectionism. In order to better account for existing research, we provide the first systematic review and meta-analysis of the relationship between multidimensional perfectionism and orthorexia, and explore factors that might explain differences in findings across studies.

### Orthorexia

Orthorexia is, broadly, considered an obsessional focus on “healthy” eating and “correct" nutrition [4]. Proposed diagnostic criteria for orthorexia was provided by Dunn and Bratman [5] based on extensive review of research, existing criteria, case studies, and available measures. The criteria include (1) compulsive behaviour and/or mental preoccupation regarding dietary practices that are thought to promote optimum health (fundamental characteristic); (2) exaggerated fear of disease, personal impurity and/or negative physical sensations, accompanied by fear and shame (emotional and physical responses to dietary transgression); and (3) escalating dietary restrictions that involve progressively more frequent and/or severe “cleanses” or partial fasts (escalation from disordered eating to pathology). Dunn and Bratman [5] also identify a range of possible impairments that can be used for diagnostic purposes such as malnutrition, severe weight loss, distress and impaired functioning in life domains (e.g., social, academic, and workplace), and a dependence of body image, self-worth identity, and/or satisfaction on compliance with healthy eating.

Orthorexia is not currently recognised as a distinct clinical eating disorder in either the Diagnostic and Statistical Manual of Mental Disorders (DSM-5-TR) or International Classification of Diseases (ICD-11). In addition, there is some disagreement in regards to the degree to which orthorexia is different to other similar disorders [6]. However, recent work examining consensus on the definition and diagnostic criteria for orthorexia among researchers and treatment specialists suggests that it is most likely a distinct mental health disorder that falls within the DSM-5 category of Feeding and Eating Disorders [7]. To justify a separate diagnosis, some of the key differences between orthorexia and anorexia identified in this work were the centrality of appearance concerns and value, explicit searches for thinness or weight/shape phobia, and goal of weight loss, that are key to anorexia but not orthorexia. Similarly, differences were suggested between orthorexia and avoidant-restrictive food intake disorder that include distinct underlying causes (aversive experiences versus worries about healthiness) and differing perceived consequences of eating behaviour (short-term effects such as choking versus long-term effects such as poorer health). On this basis, a case for the inclusion orthorexia in the DSM-5-TR and ICD-11 is steadily building.

A number of factors have been linked to the onset of orthorexia and its aetiology. In the aforementioned consensus work this included a history of other feeding and eating disorders or mental disorders, psychosomatic problems, depressive symptoms, anxiety, and emotional dysregulation [7]. Lifestyle factors such as competitive sports participation, high levels of exercise frequency, and vegan or vegetarian eating habits were also identified. Finally, social factors such as excessive influence of media, social networks, and online forums related to eating behaviours and physical appearance were included. However, almost all of these factors were considered to need additional research to better understand their role. To complicate matters, whether orthorexia precedes, coexists with, or follows other feeding and eating disorders all remain a possibility [7, 8]. Orthorexia also shares many of the associated risk factors with other feeding and eating disorders, including personality factors such as perfectionism which is the focus of this review [7].

### Multidimensional perfectionism

Perfectionism is a personality trait that is characterised by setting excessively high standards and overly critical self-evaluation [9]. It is typically regarded as multidimensional and includes various personal and interpersonal dimensions that are captured using different instruments [10]. Research has factor-analysed measures of perfectionism and found two higher-order dimensions: perfectionistic strivings and perfectionistic concerns. Perfectionistic strivings (PS) include self-oriented striving for perfection and personal standards whereas perfectionistic concerns (PC) include concern over mistakes, fear of negative evaluation by others, and feelings of discrepancy between one’s standards and one’s performance [11]. The higher-order model of perfectionism provides an organisational framework to account for and encompass different conceptual and measurement approaches to perfectionism. It also permits the examination of separate, partial, and total effects of perfectionism that can be useful to fully understand how each of the two correlated dimensions influence particular outcomes.

Perfectionism has received considerable attention in recent years with a number of meta-analytical studies now available that summarise its effects. Much of this research has focused on its relationship with psychopathology and indicators of mental health. In these cases, PC have typically shown stronger positive relationships than PS. For example, a meta-analysis of the relationship between perfectionism and depressive symptoms in longitudinal studies found that PC predicted larger increases in depressive symptoms over time than PS [12]. Similar results were found when meta-analysing the longitudinal relationship between perfectionism and anxiety symptoms [13]. These two meta-analytical studies exemplify others that suggest both PS and PC are vulnerability factors for general mental health issues, to varying degrees, with PC normally the more problematic of the two dimensions.

In regards to meta-analyses of perfectionism and eating pathology, specifically, there are two key meta-analyses. In the first meta-analysis, when examining perfectionism and psychopathology, Limburg and colleagues [14] found that both PS and PC had small-to-medium positive relationships with eating disorder symptoms—global eating pathology, binge eating, dietary restraint, drive for thinness, and thin idealisation. The analyses of anorexia and bulimia disorders were hampered by fewer studies and effect sizes but provided some tentative evidence of PC being more problematic for bulimia when examining partial effects (i.e., controlling for PS). More recently, this latter finding seems to have been confirmed by Kehayes and colleagues [15] who meta-analysed the longitudinal relationship between perfectionism dimensions and bulimic symptoms. The study found that only PC were positively related to increases in bulimic symptoms over time when baseline bulimic symptoms were controlled for. Research, to date, then, appears to justify the status of perfectionism as a transdiagnostic risk factor for eating disorders [16].

### Perfectionism and orthorexia

Consistent with the aforementioned empirical findings, there is an emerging body of research that has linked perfectionism to orthorexia. This link reflects commonality among eating disorders and the prominence of perfectionistic themes in their aetiology [17]. However, it is important to note that the link to orthorexia differs from the link to other existing disorders as a function of how perfectionism manifests. For anorexia, for instance, perfectionism manifests through irrational ideals for the body, weight, and shape [17]. In the case of orthorexia, though, perfectionism manifests in irrational nutrition and dietary practices and not necessarily beyond [7]. In addition, while adherence to rigid and strict dietary rules is common to eating disorders, the content of perfectionistic goals likely differs between obtaining “perfect weight” (anorexia and bulimia) and being “perfectly healthy” (orthorexia) [7]. As a perfectionistic goal, rigid and strict adherence to dietary rules, again, may even be an end in itself for orthorexia [18].

Researchers have recently begun to examine the relationship between PS and PC and orthorexia. Most of these studies appear to have found both PS and PC to be positively related to orthorexia (e.g., [19]). However, the size of their effects often varies. Some studies have found that PS show stronger correlations with orthorexia than PC (e.g., [20]). Other studies have found the opposite with PC showing stronger correlations than PS (e.g., [8]). There are also notable differences between studies in the strength of the observed relationships with evidence of both small effects (e.g., [21]) and large effects for PS (e.g., [22]), for example. A full and systematic account of this research is needed to survey existing research findings and to better inform researchers and practitioners of the state of knowledge in this area.

In doing so, a meta-analytical approach provides an opportunity to estimate typical effects across studies and the precision of current estimates. Meta-analysis is a useful technique that has been used extensively within the perfectionism area and has recently begun to be used to examine orthorexia (e.g., [23–25]). However, research examining perfectionism and orthorexia has yet to be the focus of this work. One of the other advantages of a meta-analytical approach is that it allows for the exploration of possible moderating factors that can account for variability between studies in effects [26]. In this way, factors that may explain why there are differences in the effect sizes across studies can be identified. This may prove especially useful for research examining orthorexia as there are notable differences between studies in regards to the sample characteristics, domains, and instruments used in existing research. We therefore also took the opportunity to explore potential moderators of the relationship between perfectionism and orthorexia in the current study.

The first moderating factor we examine is the *age* of participants. Age has been identified as a moderator in meta-analyses examining perfectionism (e.g., [27]). In that work, adults tended to display worse outcomes than adolescents (e.g., stronger relationships with negative affect). However, there is more mixed evidence of the role that age plays in orthorexia. Some studies have found that age is negatively related to orthorexia for some instruments but not others, and that age moderates the meta-analytic relations of sex differences in orthorexia with women reporting higher levels of orthorexia than men in older samples but not in younger samples [28]. In contrast, other studies have found that age does not moderate meta-analytic relations between addictive exercise behaviours and orthorexia [29]. The role of age in the perfectionism-orthorexia relationship is therefore uncertain.

The second moderating factor we examine is the *gender* of participants. Again, there is evidence that gender is a moderating factor for perfectionism. Some studies have found that gender moderates meta-analytic relations between perfectionism and personality [30]. In that work, the relationships between PS and neuroticism increased as the percentage of females in the sample increased, for example. Again, there is more mixed evidence as regard to gender in orthorexia literature. For example, some work has noted that orthorexia is higher among females [31], where other work has indicated higher levels of orthorexia in males [32]. Particularly pertinent to our study is evidence that gender moderates the relationship between perfectionism and eating disorders with a stronger relationship evident for females [33]. As such, it is possible that gender moderates the perfectionism-orthorexia relationship in the same way.

The third moderating factor we examine is the *domain* in which the study took place. There is evidence of differences between domains in regards to the strength of observed relationships of perfectionism. For example, a meta-analysis by Hill and Curran [34] identified significant differences in the strength of the relationships of PS and PC with burnout depending on whether in sport, education, and work domains. Relationships were stronger in a work domain than in sport and education. Research examining perfectionism and orthorexia has taken place in both education and sport/exercise domains but with little consideration of domain as a possible moderator (see [35]). As sport/exercise participation has been identified as a risk factor for orthorexia, studies in this domain might show, for example, stronger effects of perfectionism.

The fourth moderating factor we examined is the *instrument used to measure orthorexia*. There are a number of different instruments available to measure orthorexia. These instruments have different features and properties. Alluding to possible moderation, there is meta-analytical evidence that the instrument used affects orthorexia prevalence rates in exercising populations (e.g., [23]) and generally (e.g., [36]), with the ORTO-15 [37] related to higher prevalence rates. Some features of samples such as age also appear to be related to scores on some orthorexia instruments and not others [28]. Therefore, the instrument used to measure orthorexia is also a worthwhile candidate for a moderator in the current meta-analysis.

The final moderating factor we examine is the *instrument used to measure perfectionism*. PS and PC are often measured using different instruments and this is the case in studies examining their relationship with orthorexia. Evidence of more general differences in effects depending on perfectionism instrument is provided by Limburg and colleagues [14], among others. They found stronger effects of PS on eating psychopathology when studies used certain measures of perfectionism (e.g., F-Multidimensional Perfectionism Scale [9] versus HF-Multidimensional Perfectionism Scale [38]). It is therefore possible that the instrument used to measure perfectionism would also moderate the relationship between perfectionism and orthorexia.

### The present study

The present study aimed to provide the first systematic review and meta-analysis of research examining the relationship between multidimensional perfectionism (viz., PS and PC) and orthorexia. Based on previous research, it was hypothesised that PS would be positively related to orthorexia and PC would also be positively related to orthorexia. While there is some evidence to support possible hypotheses regarding moderating factors (age, gender, domain, and instruments), we considered these analyses exploratory.

## Methods

### Literature search and inclusion criteria

The methodological process was pre-registered on PROSPERO (CRD42022379099). The review had two stages. For the systematic review, an extensive computerised literature search was conducted following the PRISMA guidelines [39]. The following databases were searched: PsycINFO, MEDLINE, Education Abstracts, and Oxford Academic, and ScienceDirect. The search terms used were: “perfectionism” AND “orthorexia”. The search strategy included conference abstracts, reports, dissertations/thesis, and peer reviewed articles that are written in English. Reference lists were also searched. The search date was set between January 1st 1997 (the year that the term “orthorexia” was first introduced by Bratman) [40] to present. The search was conducted in April 2023. The search revealed 97 studies. The 97 studies were reviewed in full by the lead author and then subsequently checked by a co-author.

The overall aim of the search was to identify studies that included an examination of the multidimensional perfectionism-orthorexia relationship. The first step required a coder to review the search records for relevance (first author). After this step, 59 relevant articles remained. The next step involved manually removing any further duplicates, leaving 51 articles. The next step involved screening the abstracts of the remaining studies and removing studies that were unrelated to the search aims. Studies that did not include an empirical examination of multidimensional perfectionism and orthorexia were removed. For example, review papers, short communications, and articles that employed a qualitative research design were removed at this stage. This left 20 relevant studies. The remaining full text articles were further assessed for eligibility. The penultimate step involved removing any articles that included multidimensional perfectionism but no criterion variables. In the final step, the reference lists of all remaining papers were check for other relevant papers.

In total, 18^1^ eligible studies were included in the systematic review. All eligible studies (a) measured multidimensional perfectionism and orthorexia using scales that yielded quantitative values; (b) were published in English; (c) were a published journal article, thesis/dissertation, or conference presentation; and (d) included a sample that was unique (e.g., not included in both journal article and a thesis/dissertation). For the meta-analysis, additional inclusion criteria included (e) whether the study was a cross-sectional or longitudinal published study reporting of sample size and effect sizes (e.g., bivariate correlations) or information that allows them to be calculated. After full text inspection for effect sizes of the studies eligible for the systematic review, 12^2^ studies were included in the meta-analyses. See supplementary materials for the PRISMA diagram (Figure S1).

**Table 1:** Characteristics of studies included in the systematic review

				Sample					Measurement				Qual	
	Type	Country	Design	Domain	*N*	Descriptor	Age (SD)	% F	Orto	Perf	PS	PC	MQS (%)	Findings
Albery et al. [76]	Journal article	UK	Correlational / cross-sectional	Education	86	Vegan and vegetarian undergrad. and postgrad. students	33.00 (10.9)	76.7	TOS	F-MPS	–	CM	58.33	CM > healthy orthorexia (bivariate correlations) CM > orthorexia nervosa (regression)
Barnes & Caltabiano [74]	Journal article	Australia	Correlational / cross-sectional	Education	220	Undergrad. students	23.81 (8.40)	–	ORTO-15	HF-MPS	SOP	SPP	55.56	SOP, OPP, and SPP < orthorexia (bivariate correlations)
Barrada & Roncero [43]	Journal article	Spain	Correlational / cross-sectional	Education	942	Students (unspecified)	24.01 (6.4)	76.0	ORTO-15 TOS	F-MPS	–	CM	66.67	CM > healthy orthorexia and orthorexia nervosa [TOS] (bivariate correlations) CM < orthorexia [ORTO-15] (partial correlations)
Bartel et al. [19]	Journal article	Canada	Correlational / cross-sectional	Education	512	Undergrad. students	24.50	82.6	rBOT	F-MPS	PSt	CM	66.67	PTotal (including PE and PCr, PSt, CM, and DA) > orthorexia (bivariate correlations)
Brytek-Matera et al. [41] Sample 1	Journal article	Poland	Correlational / cross-sectional	Education	286	Undergrad. students	22.33 (2.38)	82.5	EHQ	F-MPS	PSt	CM	72.22	PSt > total orthorexia and knowledge; PTotal, CM, and PE + PCr > orthorexia problems (bivariate correlations)
Brytek-Matera et al. [41] Sample 2	Journal article	Italy	Correlational / cross-sectional	Education	320	Undergrad. students	21.98 (2.09)	79.0	EHQ	F-MPS	PSt	CM	72.22	PTotal, PSt, O, CM + DA, and PE + PCr > total orthorexia and orthorexia problems; PTotal, PSt, and O > orthorexia knowledge; PTotal, PSt, CM + DA, and PE + PCr > orthorexia feelings (bivariate correlations)
Domingues & Carmo [77]	Journal article	Portugal, UK, USA	Correlational / cross-sectional	Sport / exercise	469	Yoga practitioners	35–54	84.0	TOS	F-MPS	PSt	CM	41.67	PSt, CM, DA, and O > orthorexia nervosa (bivariate correlations). PSt and O > healthy orthorexia (bivariate correlations) DA > orthorexia (regression)
Hayes et al. [21]†	Journal article	US	Correlational / cross-sectional	Education	404	Undergrad. students	20.71 (4.36)	82.7	ORTO-15	F-MPS	PSt	CM	61.11	PTotal < orthorexia (bivariate correlations)
Mavrandrea & Gonidakis [31]	Journal article	Greece	Correlational / cross-sectional	Sport / exercise	241	Gym goers and CrossFit athletes	26.30	48.5	ORTO-15	APS	HS	D	72.22	HS < orthorexia (bivariate correlations). HS < orthorexia (regression)
Merhy et al. [79]	Journal article	Lebanon	Correlational / cross-sectional	Education	396	Undergrad. students	25.93 (4.96)	0	DOS	BTPS-SF	SCP	RP	72.22	SCP, RP, and NP > orthorexia (bivariate correlations) Orthorexia mediates between SCP, RP, and MDD (mediation analyses)
Miley et al. [70]	Journal article	UK	Correlational / cross-sectional	General	670	Community	39.00	88.0	E-DOS	BTPS-SF	SCP	RP	75.00	SCP, RP, and NP > orthorexia (bivariate correlations) RP > orthorexia (regression)
Myrissa et al. [73]†	Journal article	UK	Correlational / cross-sectional	Sport / exercise	215	Elite and recreational athletes	26.71 (6.83)	–	TOS EHQ	HF-MPS-SF	SOP	SPP	25.00	SOP and orthorexia knowledge > elite athletes versus recreational athletes (t-tests) OOP > healthy orthorexia [TOS] and orthorexia knowledge, orthorexia problems, and total orthorexia [EHQ] (regression)
Novara et al. [20]†	Journal article	Italy	Correlational / cross-sectional	Education	329	Undergrad. students	36.07	80.2	EHQ	F-MPS	PSt	CM	75.00	PTotal in obesity, binge eating disorder, and diet groups > orthorexia (regression)
Novara et al. [71]	Journal article	Italy	Correlational / cross-sectional	Education	302	Undergrad. students	20.71 (4.11)	53.7	EHQ	F-MPS	PSt	CM	75.00	PTotal, PSt, CM, DA, PE, PCr, and O > total orthorexia; DA, PE, and PCr > orthorexia problems; PTotal, PSt, CM, DA, PE, and O > orthorexia knowledge; PTotal, PSt, CM, DA, PE, and PCr > orthorexia feelings (bivariate correlations)
Oberle et al. [75]	Journal article	US	Correlational / cross-sectional	Education	459	Undergrad. students	19.85 (2.79)	80.0	EHQ	F-MPS	PSt	CM	63.89	PTotal, PSt, CM, O, and PE > total orthorexia; PTotal, PSt, and O > orthorexia behaviours; PTotal, PSt, CM, DA, O, PCr, and PE > orthorexia problems (partial correlations)
Osa & Calogero [22]†	Journal article	Canada	Correlational / cross-sectional	Sport / exercise	228	Physically active sportswomen	25.11 (5.70)	100	ORTO-15	S-MPS-2	PSt	CM	69.44	PSt + CM < orthorexia (bivariate correlations)
Pratt et al. [69]	Journal article	UK	Longitudinal / correlational	Sport / exercise	177	Gym goers	31.60 (7.9)	38.4	EHQ	HF-MPS-SF	SOP	SPP	72.22	OOP, OOP, PSP, NDCI > orthorexia knowledge, problems, and feelings; SOP > orthorexia knowledge (bivariate correlations) SOP > orthorexia knowledge, OOP > orthorexia problems and orthorexia feelings (regression)
Rogoza et al. [72]	Journal article	Lebanon	Correlational / cross-sectional	Education	363	Undergrad. students	22.65 (3.48)	61.7	ORTO-R TOS	BTPS-SF	SCP	RP	75.00	SCP, RP, and NP > orthorexia [ORTO-R], SCP and NP > orthorexia nervosa [TOS] (bivariate correlations)
Yayin & Ergun [78]†	Journal article	Turkey	Correlational / cross-sectional	Education	445	Undergrad. students	22.30 (3.04)	100	TOS	APS	HS	D	69.44	PTotal > orthorexia nervosa (bivariate correlations) PTotal mediates between orthorexia and maternal rejection (mediation analyses)

† = not included in meta-analysis; % F = percentage female; Orto = instrument used to measure orthorexia; Perf = instrument used to measure perfectionism; PS = perfectionistic strivings; PC = perfectionistic concerns; MQS = methodological quality score; TOS = Teruel orthorexia scale [43]; rBOT = Bratman orthorexia test-revised [68]; EHQ = Eating habits questionnaire [42]; DOS = Dusseldorf orthorexia scale [44]; E-DOS = Dusseldorf orthorexia scale-English version [67]; ORTO-15 [37]; ORTO-R [66]; HF-MPS = Hewitt-Flett Multidimensional perfectionism scale [38]; F-MPS = Frost Multidimensional perfectionism scale [9]; S-MPS-2 = Sport-multidimensional perfectionism scale-2 [49]; HF-MPS-SF = Hewitt-Flett Multidimensional perfectionism scale-short from [10]; APS = Almost perfect scale [50]; BTPS-SF = Big three perfectionism scale-short form [51]. >  = positively associated with; <  = negatively associated with; PTotal = total perfectionism score; PSt + CM = composite score [personal standards, concerns over mistakes]; SOP = self-oriented perfectionism; SPP = socially prescribed perfectionism; OOP = other-oriented perfectionism; DA = doubts about actions; PE = parental expectation; PCr = parental criticism; PE + PCr = composite score [parental expectation, parental criticism]; CM + DA = composite score [concern over mistakes, doubts about actions]; O = organisation; CM = concern over mistakes; PSt = personal standards; HS = high standards; D = discrepancy; MDD = muscle dysmorphic disorder; SCP = self-critical perfectionism; RP = rigid perfectionism; NP = narcissistic perfectionism; AN = anorexia nervosa; BN = bulimia nervosa; BED = binge eating disorder; PSP = perfectionistic self-presentation; NDCI = nondisclosure of imperfection

### Data extraction

The final identified studies were reviewed in full by the lead author and a co-author. The following information were extracted and input into an agreed characteristics table; (a) publication author and date; (b) country; (c) study type; (d) domain; (e) sample size; (f) descriptor; (g) age and standard deviation; (h) gender (percentage female); (i) instrument used to measure orthorexia; (j) instrument used to measure perfectionism; (k) indicator of perfectionistic strivings; (l) indicator of perfectionistic concerns; (m) methodological quality assessment score; and (n) main findings. Any differences in the information extracted between reviewers were resolved by consensus and directly consulting with the articles. Table 1 presents the characteristics table.

For the meta-analysis, a standardised piloted coding sheet was used to record the following information: (a) study (publication author and date); (b) domain; (c) descriptor; (d) sample size; (e) age and standard deviation; (f) gender (percentage female); (g) instrument used to measure orthorexia; (h) instrument used to measure multidimensional perfectionism; (i) indicator of perfectionistic strivings; (j) indicator of perfectionistic concerns; (k) bivariate correlations between perfectionistic strivings and perfectionistic concerns (l) bivariate and partial correlations between perfectionistic strivings and orthorexia; and (m) bivariate and partial correlations between perfectionistic concerns and orthorexia. Again, this process was undertaken by the lead author and a co-author and differences resolved by consensus and consulting the articles. See supplementary materials a summary of the characteristics of studies included in the meta-analyses (Table S2).

Measures of orthorexia included were ORTO-15 and its variants [37], Eating Habits Questionnaire (EHQ) [42], Teruel Orthorexia Scale (TOS) [43], Düsseldorfer Orthorexia Scale (DOS) [44], Bratman Orthorexia Test (BOT) [45], and Orthorexia Nervosa Inventory (ONI) [46].

Measures of multidimensional perfectionism included were: Frost Multidimensional Perfectionism Scale (F-MPS) [9], Hewitt and Flett Multidimensional Perfectionism Scale (MPS) [38], Almost Perfect Scale (APS) [47], Big Three Perfectionism Scale [48], and Sport-Multidimensional Perfectionism Scale 2 (S-MPS-2) [49]. Indicators of PS were personal standards from the F-MPS [9], self-oriented perfectionism from the HF-MPS [38], high standards from the APS [47, 50], and rigid perfectionism from the Big Three Perfectionism Scale Short Form [51]. Indicators of PC were concerns over mistakes, doubts about action, discrepancy, socially prescribed perfectionism, and self-critical perfectionism subscales from the same instruments identified above. However, no multidimensional instrument was excluded from the meta-analysis if found during the search.

For the purposes of meta-analysis, when a study reported multiple effect sizes for the perfectionism (PS and PC) and orthorexia relationship, only one effect size was used. If total orthorexia scores were reported, these were used. If there were multiple measures/subscales for perfectionism or orthorexia, or effects from multiple waves of data, we used the average of the reported effect sizes. There were two exceptions to this approach—the concerns over mistakes subscale was considered a better indicator of PC than doubts about action from the F-MPS and orthorexia nervosa subscale was considered a better indicator of orthorexia than healthy orthorexia from the TOS. We employed this approach to ensure that the effect sizes used in the analyses were independent and to prevent overrepresentation of studies that included multiple effects. Doing so, avoided artificial inflation of sample size and distortion of standard error estimates in the meta-analysis [52].

### Methodological quality appraisal

We appraised the methodological quality of each study to provide important information on the methodological adequacy (see Table 1 and supplementary materials S3). In line with previous research (e.g., [53]), we reviewed methodological characteristics and assigned each study a methodological quality score (MQS). We used specific characteristics that were based on the NICE (National Institute for Health and Care Excellence) quality appraisal checklist for quantitative studies reposting correlations and associations. The quality appraisal checklist assesses the following criteria: characteristics of study participants, definition of independent variables, outcomes assessed and methods of analyses. Responses are reported as ++ (all or most checklist criteria have been fulfilled), + (some of the checklist criteria have been fulfilled),—(few or no checklist criteria have been fulfilled), NR (not reported), and NA (not applicable).

### Meta-analytical procedures

Recommendations of Lipsey and Wilson [52] were followed when conducting the meta-analyses. Effect sizes and confidence intervals (CIs) were derived using a random-effects model. Schmidt and colleagues [54] suggested that random effects models allow for generalisation to future studies, beyond the present studies. The meta-analyses were conducted using Meta-Essentials software [55].

The analyses were based on Fisher’s [56] *Z* scale because correlation coefficients have a problematic standard error when weighted cumulative effects are derived [52]. Fisher’s [56] *Z* scale scores were converted back to correlation coefficients [52].

The weighted mean meta-analysed effects were used to calculate the total unique effect and relative weights of perfectionism. We calculated the total unique effect of perfectionism (TUE = β_PS_ + β_PC_), using the TUE Shinyapp [57] to ascertain whether the overall effect of perfectionism in orthorexia was neutral, adaptive, or maladaptive. To account for shared variance between PS and PC, we then calculated the relative weight indices using an R-based Web Tool [58].

Partial correlations were calculated to capture any unique relationships between perfectionism dimensions (residualised) and orthorexia. Partial correlations are used to examine linear associations between two variables when variability is removed from both variables. Partial correlations were calculated using Cohen and Cohen’s [59] (pp.74, Eq. 3.3.11) equation and R code [60].

Moderation was examined using the heterogeneity of the effect sizes (*Q*_*T*_), where statistically significant heterogeneity indicated possible moderation and the need for subgroup analysis. Moderation categories included participant mean age, domain, gender (percentage female), the perfectionism instrument, the orthorexia instrument, and methodological quality.

Heterogeneity was assessed by calculating the degree of inconsistency in the observed relationship across studies (*I*^*2*^). *I*^*2*^ values of 25%, 50%, and 75% were classed as being indicative of low, medium, and high levels of heterogeneity [61]. Subgroup analyses were performed where any heterogeneity exited and were centred around the heterogeneity explained by any categorization in the data (*Q*_*B*_). *Q*_*B*_ was deemed statistically significant if there are differences between effect size categories. Subgroup analyses included domain, the perfectionism instrument, and the orthorexia instrument. Subgroup analyses were analysed in line with perfectionism indicators. Specific differences were examined by comparing the overlap between 95% CIs for effect sizes [62]. Separate weighted meta-regression models were used for non-categorical moderators (e.g., age) to examine if the variables are statistically significant covariates.

Publication bias was assessed by examining Rosenthal’s [63] fail-safe number, which should be greater than 5*k* + 10 (where *k* equals the number of effect sizes) [63]. Egger’s regression intercept was then used, where the 95% CIs of Egger’s regression coefficient included zero if no publication bias is present [64]. Any asymmetry in the study distributions were corrected using trim and fill method recommended by Duval and Tweedie [65], which also provided adjusted effect sizes.

## Results

The results of the systematic review are structured around the characteristics, methodological quality, and findings of the identified studies. Within study characteristics, we report the sample demographics, orthorexia instrument, perfectionism instrument, indicators of perfectionism, and the research designs of the studies. Within methodological quality, we report the MQS and describe what higher versus lower methodological quality means. Finally, we report and evaluate the main findings of the studies.

### Study characteristics

#### Descriptive information

Twelve studies consisted of samples with mean ages in the twenties (*n* = 4874), four studies had mean ages in the thirties (*n* = 1262), one study that had a mean age of nineteen years (*n* = 459), and one study reported an age range of 35–54 years old (*n* = 469). Samples in the majority of the studies were predominately female (*n* = 5815), apart from four studies that involved mostly males (*n* = 1072). The majority of studies took place in an education setting (*k* = 12) and included participants that were undergraduate students apart from one study that included vegan and vegetarian undergraduate and postgraduate students (*n* = 86) and one study that did not specify the type of students (*n* = 942). Of the remaining six studies, five (*n* = 1330) were carried out in a sport/exercise domain. The final study (*n* = 670) was carried out in a general domain.

#### Measures of orthorexia

Four studies (*n* = 1093) used the ORTO-15 [37]. Six studies (*n* = 1873) used the Eating Habits Questionnaire (EHQ) [42] and three studies (*n* = 1000) used the Teruel Orthorexia Scale (TOS) [43]. One study (*n* = 215) used the EHQ and the TOS, one study (*n* = 942) used both the ORTO-15 and the TOS [43], and one study (*n* = 363) used both the ORTO-R [66] a revised version of the ORTO-15, and the TOS. One study (*n* = 369) used the Dusseldorf Orthorexia Scale (DOS) [44] and one study (*n* = 670) used the English version of the Dusseldorf Orthorexia Scale (E-DOS) [67]. Finally, the remaining study (*n* = 512) used the Revised-Bratman Orthorexia Test (rBOT) [68].

#### Measures of perfectionism

Nine studies (*n* = 4109) used the F-MPS [9], one of which used total scores (*n* = 404) and two (*n* = 1021) of which included only the concerns over mistakes subscale from the F-MPS. One study (*n* = 228) used composite scores from the Sport-Multidimensional Perfectionism Scale-2 (S-MPS-2) [49]—a contextualised version of on the F-MPS. One of the studies (*n* = 220) adopted the original HF-MPS [38], with two further studies (*n* = 392) using the HF-MPS-Short From (HF-MPS-SF) [10]. Finally, two studies (*n* = 686) used the Almost Perfect Scale (APS) [47, 50], one of which used total scores (*n* = 445) and three studies (*n* = 1402) used the Big Three Perfectionism Scale-Short Form (BTPS-SF) [51].

#### Indicators of perfectionism

In terms of PS, nine studies (*n* = 3309) reported personal standards as an indicator of PS, three studies (*n* = 612) used self-oriented perfectionism and three studies (*n* = 1429) used self-critical perfectionism as an indicator. Two studies (*n* = 686) reported high standards as an indicator, and two studies (*n* = 1028) only reported indicators of PC. For PC, eleven studies (*n* = 4337) used concern over mistakes as an indicator. Three studies (*n* = 612) used socially prescribed perfectionism and three studies (*n* = 1429) used rigid perfectionism as an indicator. Finally, two studies (*n* = 686) reported discrepancy as an indicator.

#### Study designs

Nearly all of the studies (*k* = 17) employed a cross-sectional/correlational design when examining the relationship between perfectionism and orthorexia. The remaining study (*k* = 1) adopted a longitudinal/correlational design and this included two-waves of data with all measures completed twice six weeks apart [69].

### Methodological quality of studies

The overall methodological quality of the studies was provided as a percentage of the maximum possible score (viz., a percentage of 36 points). Higher percentage scores reflected higher methodological quality (see Tables 1 and S2). The MQS for each study ranged from 25 to 75% (*M* = 65.2, *SD* = 12.91). Four studies received an MQS of 75% [20, 70–72], The majority of the studies (*n* = 12; one of which had two samples) received an MQS of between 55.6% and 72.2%. One study received an MQS of 41.7%. Finally, the lowest scoring study received an MQS of 25% [73]. In this case, relevant methodological information was not available as the paper was published as an abstract. As such, the scoring largely represents the absence of information as opposed to confirmed methodological quality.

### Summary of findings

Most studies examined the perfectionism-orthorexia relationship, the comparative predictive ability of different dimensions of perfectionism, or mediation effects in education and sport/exercise domains. In terms of the findings, when examining relationships, all in education, a range of dimensions of perfectionism (e.g., concerns over mistakes, doubts about actions, narcissistic perfectionism, organisation, other-oriented perfectionism, parental expectations/criticism, personal standards, rigid perfectionism, self-oriented perfectionism, socially prescribed perfectionism, and self-critical perfectionism) were found to be positively correlated with orthorexia [19, 20, 43, 72, 74, 75]. This was a consistent finding for both undergraduate and postgraduate students. Occasionally dimensions of perfectionism were unrelated to total orthorexia scores in this domain (e.g., doubts about actions and parental criticism) [75]. Findings were similar in other domains such as sport/exercise when using composite scores to measure perfectionism [22].

In regards to studies examining the predictive ability of different dimensions of perfectionism, Albery and colleagues [76] and Domingues and Carmo [77] found that, when considered alongside other dimensions of perfectionism, concerns over mistakes and doubts about actions were unique predictors of orthorexia in education (undergraduate students) and sport/exercise (yoga practitioners) domains (organisation and personal standards were not). Miley and colleagues [70] also found that rigid perfectionism was a unique predictor of orthorexia (community sample). In research supporting the importance of PS, especially, Mavrandrea and Gonidakis [31] found that high standards was a unique predictor of orthorexia in the sport/exercise domain (but discrepancy was not). In addition, in the only longitudinal study to examine the predictive ability of perfectionism, Pratt and colleagues [69] found that self-oriented perfectionism and other-oriented perfectionism predicted orthorexia over time in a sample of gym goers.

Two recent studies tested mediation models. In the first study, Yayin and Ergun [78] examined the mediating role of perfectionism in the relationship between perceived maternal rejection and orthorexia in female undergraduate students. They found that high standards and discrepancy mediated this relationship. In the second study, Merhy and colleagues [79] tested a more complex mediation model. This work examined the mediating role of eating attitudes and orthorexia in the relationship between perfectionism and muscle dysmorphia in undergraduate students. They found that orthorexia mediated the relationship between self-critical perfectionism and rigid perfectionism and muscle dysmorphia. Both studies examining mediation used cross-sectional designs.

Finally, the remaining studies focused mainly on comparing levels of orthorexia between country [41], clinical diagnosis [20], and sporting level [73]. Brytek-Matera and colleagues [41] compared orthorexia and obsessive–compulsive symptoms in Polish and Italian undergraduate student samples. They found that Italian sample with high orthorexia scores had higher levels of organisation, concerns over mistakes, and doubts about actions than the Polish sample. Novara and colleagues [20] compared differences in clinical and non-clinical samples at risk of developing orthorexia. Total perfectionism scores in anorexia and bulimia groups were higher than in obesity, dieting, binge eating disorder, and control groups (and also predicted orthorexia in obesity, binge eating, and dieting groups). Finally, Myrissa and colleagues [73] compared elite and recreational athletes in regards to perfectionism and orthorexia. They found that elite athletes had higher levels of self-oriented perfectionism and orthorexia than recreational athletes (and other-oriented perfectionism predicted orthorexia in the combined sample).

### Results of meta-analysis

#### Overall effect sizes

The overall weighted mean meta-analysed effect sizes for the relationship between PS and PC and orthorexia are presented in Table 2. PS and PC displayed a medium-to-large positive relationship with each other (*r*^+^  = 0.48; 95% CI [0.25, 0.65]). PS showed a small-to-medium positive relationship with orthorexia (*r*^+^  = 0.27; 95% CI [0.21, 0.32]). PC also showed a small-to-medium positive relationship with orthorexia (*r*^+^  = 0.25; 95% CI [0.18, 0.31]).

**Table 2:** Meta-analytical relationships between perfectionism and orthorexia across all studies

Predictor variables	*k*	*N*	*r*^+^	95% CI	*Q*_*T*_	*I*^*2*^	Fail-safe* N*	Egger’s intercept	95% CI	*k*^*TF*^	“Trim and fill” estimates *r* + [95% CI]
Perfectionistic strivings	11	3956	0.27	0.21, 0.32	25.68	61.06	424	− 1.84	− 11.47, 7.79	0	–
Perfectionistic concerns	13	4984	0.25	0.18, 0.31	47.98	74.99	332	− 1.72	− 8.98, 5.54	0	–
Partial perfectionistic strivings	6	2173	0.20	0.11, 0.28	12.08	58.60	67	1.84	− 9.47, 13.15	0	–
Partial perfectionistic concerns	6	2173	0.13	− 0.03, 0.26	35.33	85.85	3	− 16.32	− 40.93, 8.30	0	–

*k* = number of studies; *r*^+^  = weighted mean *r*; 95% CI = 95% confidence interval; *Q*_*T*_ = total heterogeneity of the weighted mean effect sizes; *I*^*2*^ = inconsistency in the observed relationship across studies; *k*^*TF*^ = number of imputed studies as part of the “trim and fill” method

After controlling for the relationship between dimensions of perfectionism, PS (*r*^+^  = 0.20; 95% CI [0.11, 0.26]) remained a significant positive predictor of orthorexia whereas PC (*r*^+^  = 0.12; 95% CI [− 0.03, 0.26]) did not (see again Table 2).

#### Total unique effect and relative weights of perfectionism

The weighted mean meta-analysed effects were used to calculate the total unique effect and relative weights of perfectionism. The total unique effect of PS and PC was medium-sized and significant (TUE = 0.35; 95% CI = 0.28, 0.42, n = 2173).

Dimensions of perfectionism explained 9% of the variance in orthorexia (*R*^*2*^_MODEL_ = 0.08). Relative weight analysis showed that PS (RW^PS^ = 0.05; 55.67%) made the larger contribution to the variance explained in the model than PC (RW^PC^ = 0.04; 44.33%).

#### Moderator analyses

An examination of total heterogeneity of the weighted mean effects sizes suggested that there was substantial moderation. Variability in the weighted mean effects exceeded the amount associated with sampling error and the total percentage of variations across studies due to heterogeneity was medium to high. Consequently, as planned, moderation analyses were conducted on age, gender (% female), domain, orthorexia instrument, and perfectionism instrument. Results are reported in Tables 3 and 4.

**Table 3:** Meta-regressions of effects on age and gender

Comparison	*k*	*N*	*r*^+^	*B*	SE	95% CI	β	*p*	*Q*_*model*_	*Q*_*residual*_
Perfectionistic strivings										
Age	10	3487	0.27	0.26	0.60	− 1.09, 1.61	0.15	0.66	0.19 [1], *p* = .66	8.08 [8],* p* = .43
Gender (% female)	10	3736	0.26	− 0.17	0.09	− 0.36, 0.03	− 0.56	0.05	3.71 [1], *p* = .05	8.17 [8], *p* = .42
Perfectionistic concerns										
Age	13	4984	0.25	0.00	0.01	− 0.01, 0.01	− 0.03	0.92	0.01 [1], *p* = .92	10.86 [10], *p* = .37
Gender (% female)	12	4764	0.25	− 0.02	0.13	− 0.31, 0.27	− 0.05	0.86	0.03 [1], *p* = .86	11.33 [10], *p* = .33

*r*^+^  = weighted mean *r*; *B* = unstandardised regression coefficient; SE = standard error; 95% CI = 95% confidence interval; β = standardised regression coefficient; *Q*_*model*_ = heterogeneity explained by any categorisation in the data; *Q*_*residual*_ = heterogeneity of the residual weighted sum of squares;

**Table 4:** Subgroup comparison of effect sizes between domain, orthorexia instrument, and perfectionism instrument

Comparison	Subgroup *k*	*r*^+^	95% CI	*Q*	*I*^*2*^	*Q*_*B*_
PS and domain						1.62 [1], *p* = .20
Education	7	0.28	0.19, 0.36	20.05	70.08	
Sport/Exercise	3	0.22	0.07, 0.36	2.27	8.04	
PS and orthorexia instrument†						2.93 [2], *p* = .23
EHQ	4	0.24	0.10, 0.37	6.45	53.49	
ORTO-15/ORTO-R	3	0.24	− 0.06, 0.40	70.37	720.88	
DOS	2	0.35	− 0.35, 0.79	3.22	68.95	
PS and perfectionism instrument						3.51 [2], *p* = .17
F-MPS	5	0.24	0.15, 0.32	6.60	39.41	
HF-MPS	2	0.31	− 0.53, 0.84	1.99	49.82	
BTPS-SF	3	0.32	0.14, 0.48	5.20	61.51	
PC domain						0.16 [1], *p* = .69
Education	9	0.26	0.20, 0.32	17.19	53.45	
Sport/Exercise	3	0.21	− 0.30, 0.63	28.59	93.00	
PC and orthorexia instrument†						19.62 [2], *p* < .001
EHQ	4	0.18	0.03, 0.32	6.91	56.60	
ORTO-15/ORTO-R	4	0.21	0.04, 0.36	10.54	75.53	
DOS	2	0.28	− 0.63, 0.87	6.63	84.91	
TOS-ON	2	0.41	0.06, 0.67	0.48	0.00	
PC and perfectionism instrument						1.83 [2], *p* = .40
F-MPS	7	0.27	0.17, 0.37	26.19	77.09	
HF-MPS	2	0.18	− 0.58, 0.78	1.74	42.61	
BTPS-SF	3	0.28	0.08, 0.46	6.63	69.84	

*r*^+^  = weighted mean *r*; 95% CI = 95% confidence interval; *Q* = weighted mean effect sizes; *I*^*2*^ = inconsistency in the observed relationship across studies; *Q*_*B*_ = heterogeneity explained by any categorisation in the data; PS = perfectionistic strivings; PC = perfectionistic concerns; EHQ = Eating habits questionnaire [42]; ORTO-15/ORTO-R = ORTO-15, or ORTO-R [37, 66]; DOS = Dusseldorf orthorexia scale or Dusseldorf orthorexia scale-English version [44, 67]; TOS = Teruel orthorexia scale [43]; F-MPS = Frost Multidimensional perfectionism scale [9]; HF-MPS = Hewitt-Flett Multidimensional perfectionism scale [38]; BTPS-SF = Big three perfectionism scale-short form [51]. † = ORTO-R correlation (not TOS-ON) was used for Barrada and Roncero [43] and Rogoza et al. [72]

#### Perfectionistic strivings

Meta-regression analyses showed that age (β = 0.15, *p* = 0.66) and gender (β = − 0.56, *p* = 0.05)^3^ did not moderate the relationship between PS and orthorexia.

Subgroup analyses showed no differences between domains (*Q*_*B*_ = 1.62 [1], *p* = 0.20), orthorexia instrument (*Q*_*B*_ = 2.93 [2], *p* = 0.23) or perfectionism instrument (*Q*_*B*_ = 0.3.51 [2], *p* = 0.17) in observed effects.

#### Perfectionistic concerns

Meta-regression analyses showed that age (β = − 0.03, *p* = 0.92) and gender (β = − 0.05, *p* = 0.87) did not moderate the relationship between PC and orthorexia.

Subgroup analyses showed that there were no differences found between domains (*Q*_*B*_ = 0.16 [1],* p* = 0.69) in observed effects. However, there was mixed evidence that the effects differed depending on orthorexia instrument. The *Q*_*B*_ was statistically significant (19.62 [3], *p* < 0.001) but CIs for estimates of effects for all instruments overlapped. There were no differences found between perfectionism instruments in observed effects (*Q*_*B*_ = 1.83 [2], *p* = 0.40).

#### Publication *bias*

Tests of publication bias were used to examine whether studies with statistically significant results were more likely to be published than studies with nonsignificant results. There was little evidence for publication bias. In all cases, fail-safe numbers exceeded recommended thresholds and trim and fill estimates did not differ from the original estimates. However, in one instance, for partial perfectionistic concerns, Egger’s regression intercept was significant (CIs excluded zero; see Table 2).

### Methodological quality

As an additional analysis not specified in the pre-registration, we examined if methodological quality moderated the relations of PS and PC with orthorexia. These results are presented in Table 5. Meta-regression analyses showed that the MSQ score did not moderate the relationship between PS and orthorexia (β = − 0.05, *p* = 0.89) but did moderate the relationship PC and orthorexia (β = − 0.58, *p* = 0.01).^4^

**Table 5:** Meta-regressive comparison of effect sizes on methodological quality

Comparison	*k*	*N*	*r*^+^	*B*	SE	95% CI	β	*p*	*Q*_*model*_	*Q*_*residual*_
Perfectionistic strivings										
MQS	11	3956	0.27	0.00	0.00	− 0.01, 0.01	− 0.05	.89	0.02 [1], *p* = .89	9.43 [9], *p* = .40
Perfectionistic concerns										
MQS	13	4984	0.25	− 0.01	0.00	− 0.01, -0.00	− 0.58	.01	6.26 [1], *p* = .01	12.32 [11]*, p* = .34

*r*^+^  = weighted mean *r*; *B* = unstandardised regression coefficient; SE = standard error; 95% CI = 95% confidence interval; β = standardised regression coefficient; *Q*_*model*_ = heterogeneity explained by any categorisation in the data; *Q*_*residual*_ = heterogeneity of the residual weighted sum of squares; MQS = methodological quality score

## Discussion

The aim of the present study was to provide a first systematic review and meta-analysis of the relationship between multidimensional perfectionism and orthorexia. As a first synthesis describing, evaluating, summarising, and analysing available empirical research, we provide novel insight into the role of perfectionism in orthorexia. Based on the findings of the systematic review, we discuss key findings and critical considerations below.

### Systematic review

The systematic review reveals this area of research to be small but growing. A large proportion of studies have been published recently (7 of 18 were published since 2022). As a consequence, there is considerable scope for work in this area and a need to continue to build sustained lines of research that extend understanding beyond establishing relationships between perfectionism and orthorexia. Based on existing research, we know that most dimensions of perfectionism are typically positively related to orthorexia, regardless of the instruments used and setting. In addition, there is tentative evidence that some dimensions are more important than others (e.g., doubts about actions versus personal standards) and that perfectionism itself may be a mediator between other risk factors and orthorexia (e.g., maternal rejection). Situating perfectionism as a possible risk factor itself, there is also some initial evidence that perfectionism is higher among clinical groups (e.g., anorexia/bulimia versus obesity/dieting). Overall, then, research has established a consistent general relationship between perfectionism and orthorexia with any findings beyond that being more tentative.

In advancing research further, one issue that may eventually impede progress is that this area is characterised by quite disparate approaches in regards to measures, samples, and settings. Studies routinely differ in the instruments used to measure orthorexia and perfectionism. As such, we can expect, at least to some degree, nuisances in the findings to reflect particular features of the instruments. For example, the degree to which instruments measure personal or interpersonal aspects of perfectionism (e.g., personal standards vs other-oriented perfectionism). As regard sample and setting, the majority of the studies examining relations in an education setting with undergraduate samples, but there is notable variability across studies in regards to other features such as gender and age. With evidence that risk to eating disorders differ across age (e.g., the onset of anorexia and the age of which it plateaus is 19–26 years versus 20–47 years for bulimia) [80], and those motives underlying eating disorders differ in regard to men and women (e.g., muscularity-oriented concerns in males versus thin idealisation in females) [81], more homogenous samples will be required to determine specificity (versus generalizability) of effects.

A further notable feature of existing research is that, like many areas, there is currently a reliance on cross-sectional designs and a lack of longitudinal research. Only one study has examined the perfectionism-orthorexia relationship over time [69]. In addition to being unable to establish temporal ordering and examine change in orthorexia, this also means efforts to examine mediating mechanisms are severely limited. This state-of-affairs compares poorly to work on perfectionism and anorexia and bulimia where longitudinal designs are much more common, and includes proper tests of mediation with three waves of data (e.g., [82]). If perfectionism is to be established as more than a correlate of orthorexia, this type of work will be needed. In addition, this work is also needed to provide more stringent tests of the mediation proposed in cross-sectional work (e.g., [83]) and to test other possible mechanisms from eating disorder research (e.g., stress) [84].

A final notable feature of research so far is the lack of work that includes orthorexia alongside anorexia and bulimia. This is surprising because orthorexia shares risk factors with other eating disorders and debate is ongoing regarding their similarities and differences [7]. This type of research is sorely needed. In context of perfectionism, this work would allow for better understanding of to what degree the relationship between perfectionism and orthorexia is explained by other eating disorders. In addition, if examined over time, developmental ordering could be better examined. There is some work of this kind already in anorexia and bulimia, such as that by Eddy and colleagues [85] who showed substantial diagnostic crossover between the two over a period of seven years. However, this work has yet to include multidimensional perfectionism and orthorexia. This type of research would have substantial conceptual and practical utility as we seek to better understand the perfectionism-orthorexia link in context of established eating disorders [7].

### Meta-analytical findings

Using a meta-analytical approach, as hypothesised, we found that both PS and PC showed small-to-medium positive correlations with orthorexia. These findings are the result of a largely consistent body of studies that implicate PS and PC in higher orthorexia across differing samples, domains, and measurement approaches. Of note, these findings are consistent with previous meta-analytical studies of perfectionism that have also highlighted the potential of both PS and PC to be related to eating disorders and eating disorder symptoms (e.g., [14]). As such, overall, the findings of the current meta-analysis appear to provide further evidence to support the designation of perfectionism as a possible transdiagnostic risk factor for eating disorders—inclusive of orthorexia.

The findings differ from previous meta-analytical work, though, in that we found evidence that PS may be more important than PC in relation to orthorexia. This was evident in the partial correlations which showed that PC was not a unique predictor of orthorexia when controlling for its relationship with PS. It was also evident in the estimate of total effects and relative weights where PS explained marginally more variance in orthorexia. These findings are at odds with general research that typically signals PC to be the more problematic of the two for other outcomes and, notably, for bulimia (e.g., [15]). This finding may signal that the striving component of perfectionism is more central to the development of obsessional nutrition than the worries (about adverse experiences of eating in avoidant-restrictive food intake disorder) and concerns (body weight/shape in anorexia) known to be key to other eating disorders. If this is the case, perhaps, idealisation—or pursuit of irrational ideals—is a better explanatory mechanism for the PS-orthorexia relationship than, as has been suggested elsewhere, efforts to alleviate perfectionistic stress which may be limited to PC [83]. It would be interesting to test these two alternative mediating mechanisms in future research.

### Exploring moderation

Evidence of moderation of the observed effects was mixed and ambiguous. Orthorexia instrument was found to moderate the PC-orthorexia relationship. However, while there was evidence for significant heterogeneity of effects between studies using different instruments, all confidence intervals overlapped and estimates were similar. There is perhaps emerging evidence that some instruments are associated with large effects with the TOS-ON providing the largest effect and the EHQ providing the smallest. It is, though, difficult to unpack this issue further with so few studies to compare. We note that in other research the instrument used to measure binge eating has been found to be important in regards to relations with perfectionism so it is possible that this is effect extends to other eating related constructs (see [86]). For now, given the small number of studies and mixed evidence, we recommend this finding is considered tentative and is examined further in future research. The moderating effects of instrument remains a possibility that researchers should be mindful of when selecting instruments and comparing effects across studies.

There were two other instances of possible moderation based on size of effects and statistical significance thresholds. These were gender for PS and orthorexia, and methodological quality for PC and orthorexia. However, closer scrutiny of these effects showed that they were dependent on single studies that had no females in the sample and provided the lowest MCQ score [77, 79]. Once these studies were excluded from the analyses, evidence for moderation diminished. Before dismissing the possibility of moderation entirely, though, we note interesting findings in this area relating to gender such as how orthorexia has been found to be significantly higher among males than females in some samples (e.g., [32]). Gender may, then, still play a role in shaping how perfectionism manifests in regards to orthorexia. Similarly, because of the importance of methodological quality, and previous evidence that methodological quality moderates the relationship between PS and eating disorder symptoms in children and adolescents (see [87]), we recommend that this issue is revisited, too, in future research. More high-quality studies (i.e., larger and prospective with control of confounding variables) will be especially valuable in exploring the role of gender and ensuring an unbiased account of the relationship between orthorexia and perfectionism.

## Strengths and limits

The findings of the systematic review and meta-analysis should be considered in light of the limitations of the review.

First, generalisability of the findings extends only to studies similar to those included in the review and meta-analysis. Studies were exclusively published in English in education and sport/exercise domains, and were all predominantly female adults. Samples that were adolescent, male, and non-English speaking countries, were either underrepresented or absent. As there is evidence that some of these features influence eating disorders and eating disorder symptoms (e.g., [88–90]) particular caution is required in applying the findings to these groups.

Second, in the case of the moderating role of the orthorexia instrument, although the heterogeneity between groups was significant, indicating that the effects are related to the instrument, the 95% CIs overlapped. This is a function of using a small number of studies and small samples producing imprecise estimates [91]. This specific finding should therefore be treated with caution, as should the examination of all other moderators in the subgroup analysis for the same reasons.

Third, the lower number of studies also influenced the calculation of partial effects and total unique effects [92]. These types of analyses require the correlations between PS and PC to be reported which was the case in less than half the studies. These analyses can easily be updated as and when these effects are made available. For now, the current analyses offer the best available estimates so will be valuable for researchers and practitioners.

Fourth, we explored a small number of moderating factors on the perfectionism-orthorexia relationship. In addition to revisiting factors identified in the current study, it would be beneficial to examine other moderators in future work. This includes moderators from other meta-analytical work in this area. For example, there is some evidence that BMI and cultural context may moderate the relationship between orthorexia and eating disorders (e.g., [25]). These factors may also be moderators of the relationships meta-analysed in the current study. When more studies are available, this possibility can be examined.

Finally, the meta-analytic review adopted the higher-order model of perfectionism (viz. PS and PC). We adopted the model to provide more reliable estimates of effect sizes while maximizing the number of usable studies in this area. However, there will also be value in examining the effects of individual subdimensions that are themselves reflective of PS and PC to varying degrees (see [93]). As more research accrues, a greater focus on individual subdimensions will provide more nuanced findings and offer further insight into the precise sources of risk to orthorexia.

## Conclusion

The current study provides the first systematic review and meta-analysis of the relationship between perfectionism and orthorexia. Across studies it was found that both PS and PC had small-to-medium, positive, significant relationships with orthorexia. However, PS was revealed to be more important when controlling for the overlap between perfectionism dimensions and examining relative weights. There was also tentative evidence that orthorexia instrument may be a moderating factor. Overall, the findings of the review show that, as is the case for other eating disorders and eating disorder symptoms, perfectionism is also typically positively related to orthorexia.

## What is already known on the subject?

Studies are emerging that suggest multidimensional perfectionism is related to orthorexia. This is the case in different contexts and samples. As such, there is reason to suspect that perfectionism may be important in the development and maintenance of orthorexia.

## What does this study add?

This study provides a first systematic review and meta-analysis of the relationship between multidimensional perfectionism and orthorexia. The findings suggest that both perfectionistic strivings and perfectionistic concerns are positively related to orthorexia. In addition, there was some evidence that measurement of orthorexia and other possible factors may moderate this relationship.

## Supplementary information

Below is the link to the electronic supplementary material.

Supplementary Material 1

## Data Availability

No datasets were generated or analysed during the current study.

## Footnotes

1 One of the studies [41] included in the systematic review provided two independent samples, providing 19 samples overall (see Table 1 and Figure S1 and in the Electronic Supplementary Material).

2 One study [41] included two independent samples, providing 13 samples overall for meta-analysis (see Tables S1 and S2 in the Electronic Supplementary Material).

3 This finding is heavily influenced by one study [79] which had no female participants. In the absence of this study, the null effect of gender is clearer (*k* = 10, *n* = 3,560, *r*^+^  = 0.25, B = 0.00, SE = 0.14, 95% CI = − 0.33, 0.33,* β* = 0.00, *p* = .99, *Q*_*model*_ = 0.00 [1], *p* = .99,* Q*_*residual*_ = 7.20 [7], *p* = .41.

4 This finding is heavily influenced by one study [77], which had the lowest MCQ score and the largest effect size in the analysis. In the absence of this study, the effect is smaller and not statistically significant (*k* = 12, *n* = 4,515, *r*^+^  = 0.23, B = 0.00, SE = 0.00, 95% CI = − 0.01, 0.01,* β* = − 0.24, *p* = 0.41, *Q*_*model*_ = 0.69 [1], *p* = 0.41*, Q*_*residual*_ = 11.07 [10], *p* = 0.35.
