# Supplementary material for: Multidimensional perfectionism and orthorexia: a systematic review and meta-analysis
Source: Eat Weight Disord. 2024 Oct 10;29(1):67. doi: 10.1007/s40519-024-01695-z (PMC11467028; doi:10.1007/s40519-024-01695-z)
Supplement: Supplementary file 1 — Supplementary Material 1 [file 40519_2024_1695_MOESM1_ESM.docx]

**Figure S1.** PRISMA diagram outlining search process.

**Identification of new studies via databases and registers**

Records identified from databases: (n = 97)

Records removed *before screening*: (n = 0)

**Screening**

**Identification**

Records screened in full by lead author and checked by co-author: (n = 97)

Records excluded:

Records not relevant: (n = 38)

Remaining duplicates removed: (n = 8)

Records excluded:

Records unrelated to search aims: (n = 31)

Reports sought for retrieval and abstracts screened: (n = 51)

Reports excluded:

No measure of orthorexia: (n = 2)

Reports assessed for eligibility in full: (n = 20)

Studies included in the *systematic review* (n = 18). *Note*. one study (n = 1) included two independent samples, providing 19 samples overall.

**Included**

Reports excluded:

No bivariate correlation reported: (n = 2)

Total perfectionism score used: (n = 3)

Studies included in meta-analysis (n = 12). *Note*. one study (n = 1) included two independent samples, providing 13 samples overall.

**Table S2**. Characteristics of studies included in the meta-analysis.

|  |  |  |  |  |  |  |  |  |  | Effect Sizes | | | | | | |
| --- | --- | --- | --- | --- | --- | --- | --- | --- | --- | --- | --- | --- | --- | --- | --- | --- |
|  | Sample | | | | | Measurement | | | | PS-PC | PS-O | | PC-O | | | |
| Study | Domain | Descriptor | *N* | Age (SD) | % Female | Ortho | Perf | PS | PC | *r* | *r* | *r_Partial_* | | *r* | *r_Partial_* |  |
| Albery et al. [76] | Education | Vegan and vegetarian undergrad. and postgrad. students | 86 | 33.00 (10.9) | 76.7% | TOS-ON | F-MPS | - | CM | NR | - | - | | .35 | - |  |
| Barnes & Caltabiano [74] | Education | Undergrad. students | 220 | 23.81 (8.40) | - | ORTO-15 | HF-MPS | SOP | SPP | .47 | .37 | .30 | | .24 | .08 |  |
| Barrada & Roncero [43]† | Education | Students (unspecified) | 942 | 24.01 (6.4) | 76.0% | ORTO-15  TOS-ON | F-MPS | - | CM | NR | - | - | | .30 | - |  |
| Bartel et al. [19] | Education | Undergrad. students | 512 | 24.50 | 82.6% | rBOT | F-MPS | PSt | CM | NR | .22 | - | | .25 | - |  |
| Brytek-Matera et al. [41] Sample 1 | Education | Undergrad. students | 286 | 22.33 (2.38) | 82.5% | EHQ | F-MPS | PSt | CM | NR | .15 | - | | .09 | - |  |
| Brytek-Matera et al. [41] Sample 2 | Education | Undergrad. students | 320 | 21.98 (2.09) | 79.7% | EHQ | F-MPS | PSt | CM | NR | .21 | - | | .21 | - |  |
| Domingues & Carmo [77] | Sport / exercise | Yoga practitioners | 469 | 35-54 | 84.0% | TOS-ON | F-MPS | PSt | CM | .43 | .25 | .08 | | .42 | .36 |  |
| Mavrandrea & Gonidakis [31] | Sport / exercise | Gym goers and CrossFit athletes | 241 | 26.30 | 48.5% | ORTO-15 | APS | HS | D | .17 | .14 | .13 | | .07 | .05 |  |
| Merhy et al. [79] | Education | Undergrad. Student | 396 | 25.93 (4.96) | 0.00% | DOS | BTPS-SF | RP | SCP | .72 | .40 | .22 | | .36 | .11 |  |
| Miley et al. [70] | General | Community | 670 | 39.00 | 88.0% | E-DOS | BTPS-SF | RP | SCP | .37 | .30 | .24 | | .21 | .11 |  |
| Novara et al. [71] | Education | Undergrad. students | 302 | 20.71 (4.11) | 53.7% | EHQ | F-MPS | PSt | CM | NR | .34 | - | | .28 | - |  |
| Pratt et al. [69] | Sport / exercise | Gym goers | 177 | 27.70 (9.61) | 38.4% | EHQ | HF-MPS | SOP | SPP | .58 | .24 | .22 | | .11 | -.04 |  |
| Rogoza et al. [72]† | Education | Undergrad. students | 363 | 22.65 (3.48) | 61.7% | ORTO-R  TOS-ON | BTPS-SF | RP | SCP | - | .26 | - | | .27 | - |  |

*Note.* NR = not reported; % F = percentage female; Orto = instrument used to measure orthorexia; Perf = instrument used to measure perfectionism; PS = perfectionistic strivings; PC = perfectionistic concerns; O = orthorexia; *r* = bivariate correlation; *r_Partial_* _=_ partial correlation coefficient; TOS-ON = Orthorexia nervosa subscale of the Teruel orthorexia scale [43]; rBOT = Bratman orthorexia test-revised [68]; EHQ = Eating habits questionnaire [42]; DOS = Dusseldorf orthorexia scale [44]; E-DOS = Dusseldorf orthorexia scale-English version [67]; ORTO-15 [37]; ORTO-R [66]; HF-MPS = Hewitt-Flett Multidimensional perfectionism scale [38]; F-MPS = Frost Multidimensional perfectionism scale [9]; S-MPS-2 = Sport-multidimensional perfectionism scale-2 [49]; HF-MPS-SF = Hewitt-Flett Multidimensional perfectionism scale-short from [10]; APS = almost perfect scale [50]; BTPS-SF = Big three perfectionism scale-short form [51]; SOP = self-oriented perfectionism; SPP = socially prescribed perfectionism; CM = concern over mistakes perfectionism; PSt = personal standards perfectionism; HS = high standards perfectionism; D = discrepancy; SCP = self-critical perfectionism; RP = rigid perfectionism; PS-PC = correlation between perfectionistic strivings and perfectionistic concerns; PS-O = correlation between perfectionistic strivings and orthorexia; PC-O is correlation between perfectionistic concerns and orthorexia; † = average correlation of multiple orthorexia measures/subscales reported.

**Table S3.** Quality assessment of included studies.

| Study | 1.1 | 1.2 | 1.3 | 2.1 | 2.2 | 2.3 | 2.4 | 3.1 | 3.2 | 3.3 | 3.4 | 3.5 | 4.1 | 4.2 | 4.3 | 4.6 | 5.1 | 5.2 | Total |
| --- | --- | --- | --- | --- | --- | --- | --- | --- | --- | --- | --- | --- | --- | --- | --- | --- | --- | --- | --- |
| Albery et al. [75] | + | ++ | ++ | + | ++ | NA | ++ | ++ | - | + | NA | NA | - | ++ | + | ++ | + | ++ | 21 |
| Barnes & Caltabiano [74] | + | ++ | + | + | ++ | NA | + | + | + | + | NA | NA | + | ++ | + | ++ | + | ++ | 20 |
| Barrada & Roncero [43] | ++ | ++ | ++ | ++ | ++ | NA | + | ++ | + | + | NA | + | + | ++ | + | + | + | ++ | 24 |
| Bartel et al. [19] | ++ | ++ | + | ++ | ++ | NA | ++ | ++ | + | + | NA | NA | + | ++ | ++ | + | + | ++ | 24 |
| Brytek-Matera et al. [41] data 1 | ++ | ++ | ++ | ++ | ++ | NA | ++ | ++ | + | + | NR | NA | + | ++ | + | ++ | + | + | 26 |
| Brytek-Matera et al. [41] data 2 | ++ | ++ | ++ | ++ | ++ | NA | ++ | ++ | + | + | NR | NA | + | ++ | + | ++ | + | + | 26 |
| Domingues & Carmo [77] | ++ | ++ | + | + | + | NA | - | - | + | + | NA | NA | + | ++ | - | + | + | + | 15 |
| Hayles et al. [21] | ++ | ++ | + | ++ | ++ | NA | + | ++ | + | + | NA | NA | + | ++ | + | + | + | ++ | 22 |
| Mavrandrea & Gonidakis [31] | ++ | ++ | ++ | ++ | ++ | NA | ++ | + | ++ | + | NA | NA | ++ | ++ | ++ | + | + | ++ | 26 |
| Merhy et al. [79] | ++ | + | ++ | + | ++ | NA | ++ | ++ | ++ | + | NA | NA | ++ | ++ | ++ | ++ | ++ | + | 26 |
| Miley et al. [70] | ++ | ++ | ++ | ++ | ++ | NA | ++ | ++ | ++ | + | NA | NA | ++ | ++ | + | ++ | + | ++ | 27 |
| Myrissa et al. [73] | + | + | NR | NR | - | NA | + | NR | NR | + | NA | NA | + | ++ | + | + | - | - | 9 |
| Novara et al. [20] | + | ++ | ++ | ++ | ++ | NA | ++ | ++ | + | ++ | ++ | NA | + | ++ | ++ | ++ | + | + | 27 |
| Novara et al. [71] | ++ | ++ | ++ | ++ | ++ | NA | ++ | ++ | + | + | ++ | NA | + | ++ | + | ++ | + | ++ | 27 |
| Oberle et al. [75] | ++ | ++ | + | + | ++ | NA | ++ | ++ | + | + | NA | NA | + | ++ | + | ++ | + | ++ | 23 |
| Osa & Calogero [22] | ++ | ++ | ++ | ++ | ++ | NA | ++ | + | ++ | + | NA | NA | + | ++ | + | ++ | + | ++ | 25 |
| Pratt et al. [69] | ++ | ++ | ++ | ++ | ++ | NA | + | ++ | + | + | NA | + | + | ++ | ++ | ++ | + | ++ | 26 |
| Rogoza et al. [72] | ++ | ++ | ++ | + | ++ | NA | + | ++ | ++ | ++ | NA | NA | - | ++ | ++ | ++ | ++ | + | 27 |
| Yayin & Ergun [78] | ++ | ++ | + | + | ++ | NA | + | ++ | ++ | + | NA | NA | ++ | ++ | ++ | ++ | + | + | 25 |

*Note.* ++ = minimized risk of bias. + = may not have addressed all sources of bias. - = significant sources of bias may persist. NR = Not reported. NA = Not applicable. ^1.1^Population well described. ^1.2^Population is representative. ^1.3^Selected participants represent eligible population. ^2.1^Selection bias minimised. ^2.2^Theoretical basis. ^2.3^Contamination low. ^2.4^Confounding factors identified. ^3.1^Outcome measures reliable? ^3.2^Outcome measurements complete? ^3.3^All important outcomes assessed? ^3.4^Similar follow-up time in exposure and comparison group? ^3.5^Follow-up time meaningful? ^4.1^Power analysis. ^4.2^Multiple explanatory variables considered? ^4.3^Analytical methods appropriate? ^4.4^Precision. ^5.1^Are the results valid? ^5.2^Are the findings generalisable? [Criteria ^2.5^(Setting applicable to the UK) was removed from the checklist].

**Table S4 (additional).** PRISMA 2020 Checklist.

| **Section and Topic** | **Item #** | **Checklist item** | **Location where item is reported** |
| --- | --- | --- | --- |
| **TITLE** | | |  |
| Title | 1 | Identify the report as a systematic review. | Title page 1 |
| **ABSTRACT** | | |  |
| Abstract | 2 | See the PRISMA 2020 for Abstracts checklist. | Page 2 |
| **INTRODUCTION** | | |  |
| Rationale | 3 | Describe the rationale for the review in the context of existing knowledge. | Pages 6-10 |
| Objectives | 4 | Provide an explicit statement of the objective(s) or question(s) the review addresses. | Page 10 |
| **METHODS** | | |  |
| Eligibility criteria | 5 | Specify the inclusion and exclusion criteria for the review and how studies were grouped for the syntheses. | Pages 10-12 |
| Information sources | 6 | Specify all databases, registers, websites, organisations, reference lists and other sources searched or consulted to identify studies. Specify the date when each source was last searched or consulted. | Pages 10-12 |
| Search strategy | 7 | Present the full search strategies for all databases, registers and websites, including any filters and limits used. | Pages 10-12 |
| Selection process | 8 | Specify the methods used to decide whether a study met the inclusion criteria of the review, including how many reviewers screened each record and each report retrieved, whether they worked independently, and if applicable, details of automation tools used in the process. | Pages 10-12 |
| Data collection process | 9 | Specify the methods used to collect data from reports, including how many reviewers collected data from each report, whether they worked independently, any processes for obtaining or confirming data from study investigators, and if applicable, details of automation tools used in the process. | Page 10-12 |
| Data items | 10a | List and define all outcomes for which data were sought. Specify whether all results that were compatible with each outcome domain in each study were sought (e.g. for all measures, time points, analyses), and if not, the methods used to decide which results to collect. | Pages 10-12 |
|  | 10b | List and define all other variables for which data were sought (e.g. participant and intervention characteristics, funding sources). Describe any assumptions made about any missing or unclear information. | Pages 10-12 |
| Study risk of bias assessment | 11 | Specify the methods used to assess risk of bias in the included studies, including details of the tool(s) used, how many reviewers assessed each study and whether they worked independently, and if applicable, details of automation tools used in the process. | Pages 10-13 |
| Effect measures | 12 | Specify for each outcome the effect measure(s) (e.g. risk ratio, mean difference) used in the synthesis or presentation of results. | Pages 14-15 |
| Synthesis methods | 13a | Describe the processes used to decide which studies were eligible for each synthesis (e.g. tabulating the study intervention characteristics and comparing against the planned groups for each synthesis (item #5)). | Pages 11-16, Tables 1, S1, S2, & S3 |
|  | 13b | Describe any methods required to prepare the data for presentation or synthesis, such as handling of missing summary statistics, or data conversions. | Pages 11-13, Figure S1 |
|  | 13c | Describe any methods used to tabulate or visually display results of individual studies and syntheses. | Tables 1, S1, & S2 |
|  | 13d | Describe any methods used to synthesize results and provide a rationale for the choice(s). If meta-analysis was performed, describe the model(s), method(s) to identify the presence and extent of statistical heterogeneity, and software package(s) used. | Pages 15-15 |
|  | 13e | Describe any methods used to explore possible causes of heterogeneity among study results (e.g. subgroup analysis, meta-regression). | Pages 14-15 |
|  | 13f | Describe any sensitivity analyses conducted to assess robustness of the synthesized results. | Pages 14-15 |
| Reporting bias assessment | 14 | Describe any methods used to assess risk of bias due to missing results in a synthesis (arising from reporting biases). | Pages 14-15 |
| Certainty assessment | 15 | Describe any methods used to assess certainty (or confidence) in the body of evidence for an outcome. | Pages 14-15 |
| **RESULTS** | | |  |
| Study selection | 16a | Describe the results of the search and selection process, from the number of records identified in the search to the number of studies included in the review, ideally using a flow diagram. | Figure 1 |
|  | 16b | Cite studies that might appear to meet the inclusion criteria, but which were excluded, and explain why they were excluded. | Table 1 & S2 |
| Study characteristics | 17 | Cite each included study and present its characteristics. | Pages 16-20 |
| Risk of bias in studies | 18 | Present assessments of risk of bias for each included study. | Page 22, Table S2 |
| Results of individual studies | 19 | For all outcomes, present, for each study: (a) summary statistics for each group (where appropriate) and (b) an effect estimate and its precision (e.g. confidence/credible interval), ideally using structured tables or plots. | Tables S2, 2, 3, 4, & 5 |
| Results of syntheses | 20a | For each synthesis, briefly summarise the characteristics and risk of bias among contributing studies. | Page 22, Table S2 |
|  | 20b | Present results of all statistical syntheses conducted. If meta-analysis was done, present for each the summary estimate and its precision (e.g. confidence/credible interval) and measures of statistical heterogeneity. If comparing groups, describe the direction of the effect. | Pages 20-22, Tables 2, 3, 4, & 5 |
|  | 20c | Present results of all investigations of possible causes of heterogeneity among study results. | Pages 20-22 |
|  | 20d | Present results of all sensitivity analyses conducted to assess the robustness of the synthesized results. | Pages 20-22 |
| Reporting biases | 21 | Present assessments of risk of bias due to missing results (arising from reporting biases) for each synthesis assessed. | Pages 20-22 |
| Certainty of evidence | 22 | Present assessments of certainty (or confidence) in the body of evidence for each outcome assessed. | Pages 20-22 |
| **DISCUSSION** | | |  |
| Discussion | 23a | Provide a general interpretation of the results in the context of other evidence. | Pages 22-27 |
|  | 23b | Discuss any limitations of the evidence included in the review. | Pages 22-28 |
|  | 23c | Discuss any limitations of the review processes used. | Pages 27-28 |
|  | 23d | Discuss implications of the results for practice, policy, and future research. | Pages 27-28 |
| **OTHER INFORMATION** | | |  |
| Registration and protocol | 24a | Provide registration information for the review, including register name and registration number, or state that the review was not registered. | Page 10 |
|  | 24b | Indicate where the review protocol can be accessed, or state that a protocol was not prepared. | Page 10 |
|  | 24c | Describe and explain any amendments to information provided at registration or in the protocol. | N/A |
| Support | 25 | Describe sources of financial or non-financial support for the review, and the role of the funders or sponsors in the review. | - |
| Competing interests | 26 | Declare any competing interests of review authors. | - |
| Availability of data, code and other materials | 27 | Report which of the following are publicly available and where they can be found: template data collection forms; data extracted from included studies; data used for all analyses; analytic code; any other materials used in the review. | - |
